# Bioinformatic Analysis of Correlation between Immune Infiltration and COVID-19 in Cancer Patients

**DOI:** 10.7150/ijbs.48639

**Published:** 2020-07-06

**Authors:** Xin Huang, Chaobin He, Xin Hua, Anna Kan, Shuxin Sun, Jun Wang, Shengping Li

**Affiliations:** 1 Sun Yat-sen University Cancer Center, State Key Laboratory of Oncology in South China, Collaborative Innovation Center for Cancer Medicine, Guangzhou 510060, China.; 2 Department of Experimental Research, Sun Yat-sen University Cancer Center, Guangzhou 510060, China.; 3 Department of Pancreatobiliary Surgery, Sun Yat-sen University Cancer Center, Guangzhou 510060, China.; 4 Department of Medical Oncology, Sun Yat-sen University Cancer Center, Guangzhou 510060, China.; 5 Department of Hepatic Surgery, Sun Yat-sen University Cancer Center, Guangzhou 510060, China.

**Keywords:** ACE2, COVID-19, immune infiltration, cancer

## Abstract

In 2020, severe acute respiratory syndrome coronavirus 2 (SARS-CoV-2) has caused infections worldwide. However, the correlation between the immune infiltration and coronavirus disease 2019 (COVID-19) susceptibility or severity in cancer patients remains to be fully elucidated. ACE2 expressions in normal tissues, cancers and cell lines were comprehensively assessed. Furthermore, we compared ACE2 expression between cancers and matched normal tissues through Gene Expression Profiling Interactive Analysis (GEPIA). In addition, we performed gene set enrichment analysis (GSEA) to investigate the related signaling pathways. Finally, the correlations between ACE2 expression and immune infiltration were investigated via Tumor Immune Estimation Resource (TIMER) and GEPIA. We found that ACE2 was predominantly expressed in both adult and fetal tissues from the digestive, urinary and male reproductive tracts; moreover, ACE2 expressions in corresponding cancers were generally higher than that in matched healthy tissues. GSEA showed that various metabolic and immune-related pathways were significantly associated with ACE2 expression across multiple cancer types. Intriguingly, we found that ACE2 expression correlated significantly with immune cell infiltration in both normal and cancer tissues, especially in the stomach and colon. These findings proposed a possible fecal-oral and maternal-fetal transmission of SARS-CoV-2 and suggested that cancers of the respiratory, digestive or urinary tracts would be more vulnerable to SARS-CoV-2 infection.

## Introduction

Severe acute respiratory syndrome coronavirus 2 (SARS-CoV-2) belongs to the beta genus coronavirus 1 and has caused an outbreak of coronavirus diseases 2019 (COVID-19) worldwide in 2020. As of 22 June 2020, a total of 9044581 cases have been identified around the world (https://www.worldometers.info/coronavirus/), and SAR-CoV-2 has been declared a pandemic by the World Health Organization (WHO).

As the functional receptor for the spike glycoprotein of SARS-CoV-2, angiotensin-converting enzyme 2 (ACE2) has played a crucial role in SARS-CoV-2 infection 2. Epithelial cells that have ACE2 expression in normal lungs are the main target of SARS-CoV-2 3. In addition, others have found ACE2 mRNA and protein expression in renal, cardiovascular and gastrointestinal tissues 4, 5; the conjunctivae, digestive and urogenital tracts are also exposed to the external environment, providing potential routes of transmission. Likewise, other studies have found SARS-CoV-2 RNA in feces, urine and gastrointestinal mucosa 6.

Both humoral and cellular immunity participate in the protection against SARS-CoV-2 infection 7. Evidence has indicated that the dysregulation of the immune response, especially T cells, might be highly involved in the pathological process of COVID-19 8. Meanwhile, others also demonstrated that aberrant and excessive immune cells, such as monocytes and macrophages, played an immune damaging role in COVID-19 9. By postmortem biopsies, others researchers have found intestinal infiltration of mononuclear cells in the lungs and indicated that overwhelming inflammation and cytokine-associated lung injury could be important in the progression of COVID-19 3, 9, 10.

Cancer is associated with immune dysfunction 11, and cancer patients are more prone to infections because of the systemic immunosuppressive state caused by malignancy or anticancer treatments, such as chemotherapy or surgery 12. Moreover, accumulating evidences have demonstrated that cancer is closely associated with chronic inflammation, and the tumor microenvironment (TME) is infiltrated by various blood-derived immune cells 13. Liang et al. found that cancer patients had a higher risk of COVID-19 and a poorer prognosis than those without cancer 14. Another study reported that cancer patients had an increased risk of COVID-19 compared with the general population 15. However, it is worth noting that the association between COVID-19 and cancer remains unknown due to the small sample size and high heterogeneity of the cancer patients in these studies 16.

ACE2 has been reported to inhibit cancer progression in liver hepatocellular carcinoma (LIHC) 17 and pancreatic adenocarcinoma (PAAD) 18, whereas opposite results were observed in kidney renal clear cell carcinoma (KIRC) 19. However, the correlations among SARS-CoV-2 infection, ACE2 expression and immune cell infiltration in tumor tissues, especially lung cancer and gastrointestinal cancers, have not been fully elucidated. In this study, we aimed to investigate the ACE2 expression in cancer patients or healthy individuals through bioinformatics analysis.

## Methods

### Gene expression analysis

ACE2 expression in normal and cancer tissues was assessed by the Human Protein Atlas (HPA) (http://www.proteinatlas.org/) 20, which has now incorporated baseline expression profiles of tissues from the Genotype-Tissue Expression (GTEx) and FANTOM5 projects; and ACE2 expression in human cancer cell lines was determined through the Cancer Cell Line Encyclopedia (CCLE) (https://portals.broadinstitute.org/ccle) 21. In addition, we used data from GTEx to compare ACE2 expression between male and female with R package ggpubr. Finally, ACE2 expression in single cells was explored through the human cell landscape (HCL) (http://bis.zju.edu.cn/HCL/) constructed by Guo et al. 22, including both fetal and adult tissues.

### Comparisons of ACE2 expression among cancer, adjacent normal tissue and healthy tissue

Gene Expression Profiling Interactive Analysis (GEPIA) is an interactive website that provides customizable functions, including differential expression analysis, profiling plotting and correlation analysis based on the RNA-Seq expression data of 9736 tumors and 8587 normal samples from The Cancer Genome Atlas (TCGA) and GTEx datasets (http://gepia2.cancer-pku.cn/) 23. It was used to compare ACE2 expression between cancer and normal tissues. In addition, we downloaded the data of number of fragments per kilobase of exon per million reads (FPKM) in cancer, adjacent normal tissue and healthy tissue from TCGA or GTEx, which were normalized in R environment using NormalizeBetweenArrays 24. In addition, pairwise comparisons of ACE2 expression were performed in TCGA datasets.

### Gene set enrichment analysis (GSEA)

Genes and Kyoto Encyclopedia of Genes and Genomes (KEGG) pathways associated with ACE2 expression across various types of cancer were analyzed by GSEA as previously described by using the R package clusterProfiler 25, with the normalized enrichment score (NES) calculated.

### Prognostic value analysis

The relationships between the gene expression level of ACE2 and the overall survival (OS) or disease-free survival (DFS) of cancer patients were analyzed using GEPIA and the log-rank test, with Cox proportional hazard ratio (HR) and 95% confidence intervals calculated.

### Correlation analysis of gene expression

Tumor Immune Estimation Resource (TIMER) is a comprehensive web server for the systematic analysis of immune infiltration across diverse cancer types (http://timer.cistrome.org/) 26, as described previously 27.

Furthermore, we explored the correlation between gene expression and tumor stage through GEPIA, and gene expression correlation analysis was performed for the given TCGA and GTEx datasets in GEPIA, with the Spearman method being used.

### Statistical analysis

For comparisons among cancer, adjacent normal tissue and healthy tissue, Kruskal-Wallis tests with post hoc using Dunn's method, Wilcoxon matched-pairs signed rank tests or paired t tests were performed (GraphPad Prism 7.0 software). Two-side p < 0.05 was considered statistically significant, unless otherwise specified.

## Results

### ACE2 was predominantly expressed by glandular cells of the digestive, urinary and male reproductive tracts in both the fetus and the adult

**Figure 1:**
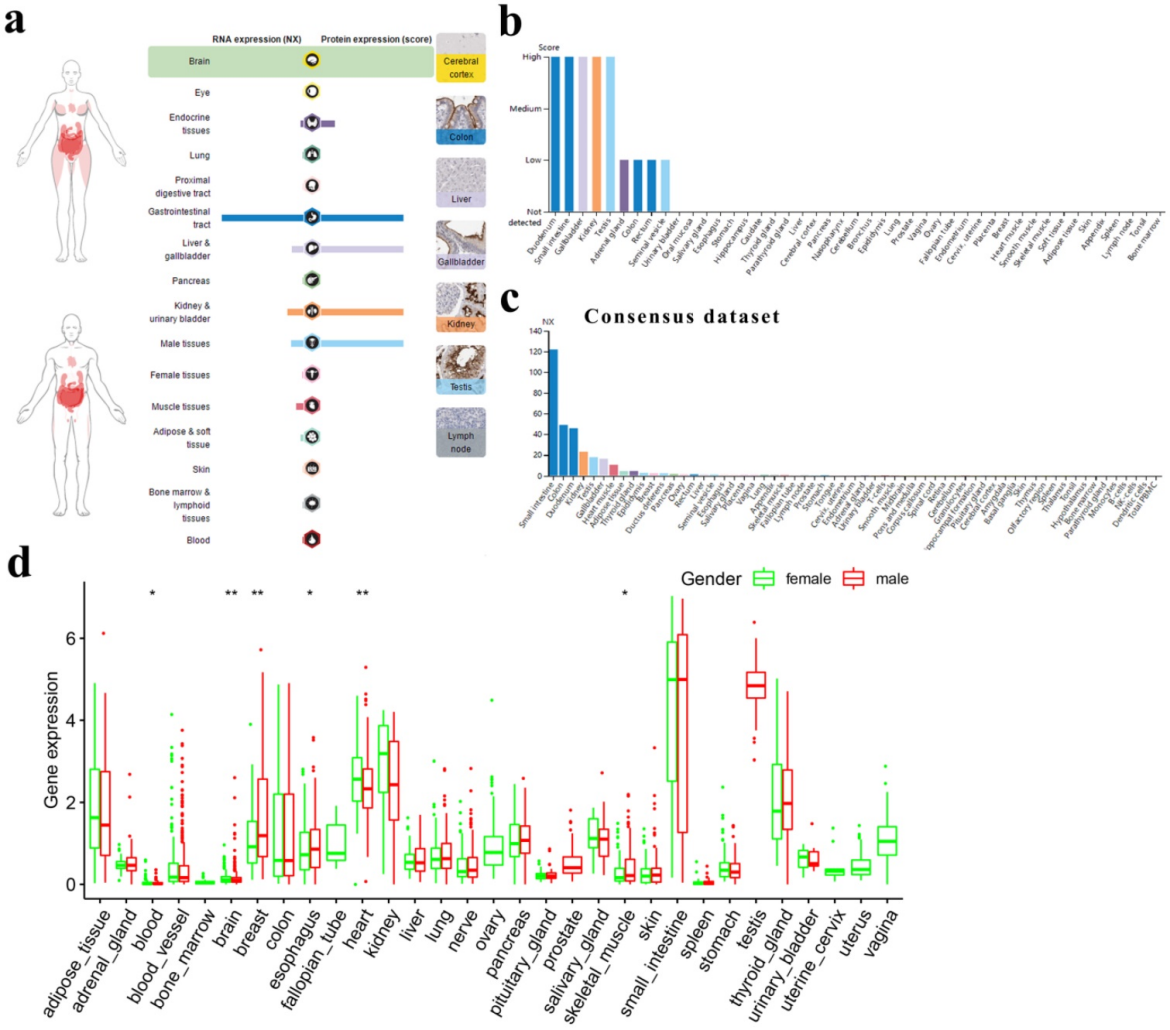
** ACE2 expression in normal human tissues.** (**a**) Overview of RNA and protein expression data in the human body. Left panel: the depth of red represents the different expression levels. Middle panel: bars represent the highest NX (RNA expression) and expression score (protein expression) found in a particular group of tissues. Right panel: representative images of immunohistochemical staining for ACE2 in the normal cerebral cortex, colon, liver, gallbladder, kidney, testis and lymph node. (**b**) Protein expressions of ACE2 in normal tissues, which were ranked by the expression levels. The y-axis represents the scores based on immunohistochemistry. (**c**) RNA expressions of ACE2 in normal tissues, which were ranked by the expression levels. The data were from the consensus dataset based on a combination of all three sources (HPA RNA-seq data, GTEx RNA-seq data and FANTOM5 data). (d) Difference in ACE2 expression between men and women from GTEx data. The y-axis represents transformed log2(FPKM+1). NX, consensus normalized expression; HPA, Human Protein Atlas; GTEx, Genotype-Tissue Expression; FPKM, number of fragments per kilobase of exon per million reads. *, P < 0.05; **, P < 0.01.

Fig. 1a shows the overview of ACE2 expression by human subjects from the HPA database. We found that ACE2 expression in the digestive, urinary and male reproductive tracts was higher than that in other tissues, at both the RNA and protein levels.

For the digestive tract, the immunohistochemical results showed that the duodenum, small intestine and gallbladder expressed ACE2 at high levels; the colon and rectum expressed ACE2 at medium levels; and the oral mucosa, salivary gland, esophagus, stomach, liver and pancreas expressed almost no ACE2. For the urinary and male reproductive tracts, we found that the kidney and testis expressed high levels of ACE2; the seminal vesicle expressed low levels of ACE2; and the urinary bladder, epididymis and prostate expressed almost no ACE2 (Fig. 1b). A similar trend was observed for mRNA expression, with consensus expression in the small intestine, colon, duodenum, kidney and testis ranking in the top 5 (Fig. 1c). In general, there was no big difference in ACE2 expression between men and women across different tissues (Fig. 1d).

Finally, we found that ACE2 expression was mainly restricted to glandular cells and Leydig cells (Table S1) and that peripheral blood cells expressed almost no ACE2 (Fig. S1). Moreover, by analyzing single-cell RNA-seq data through HCL, we found that ACE2 was a marker gene for hepatocytes/endodermal cells and enterocytes. Meanwhile, fetal enterocytes, epithelial cells, proximal tubule progenitors and goblet cells expressed ACE2 at relatively high levels, while fibroblasts and epithelial cells expressed ACE2 at relatively low levels (Table 1).

### Differential expression of ACE2 between cancer and matched normal tissues

**Figure 2:**
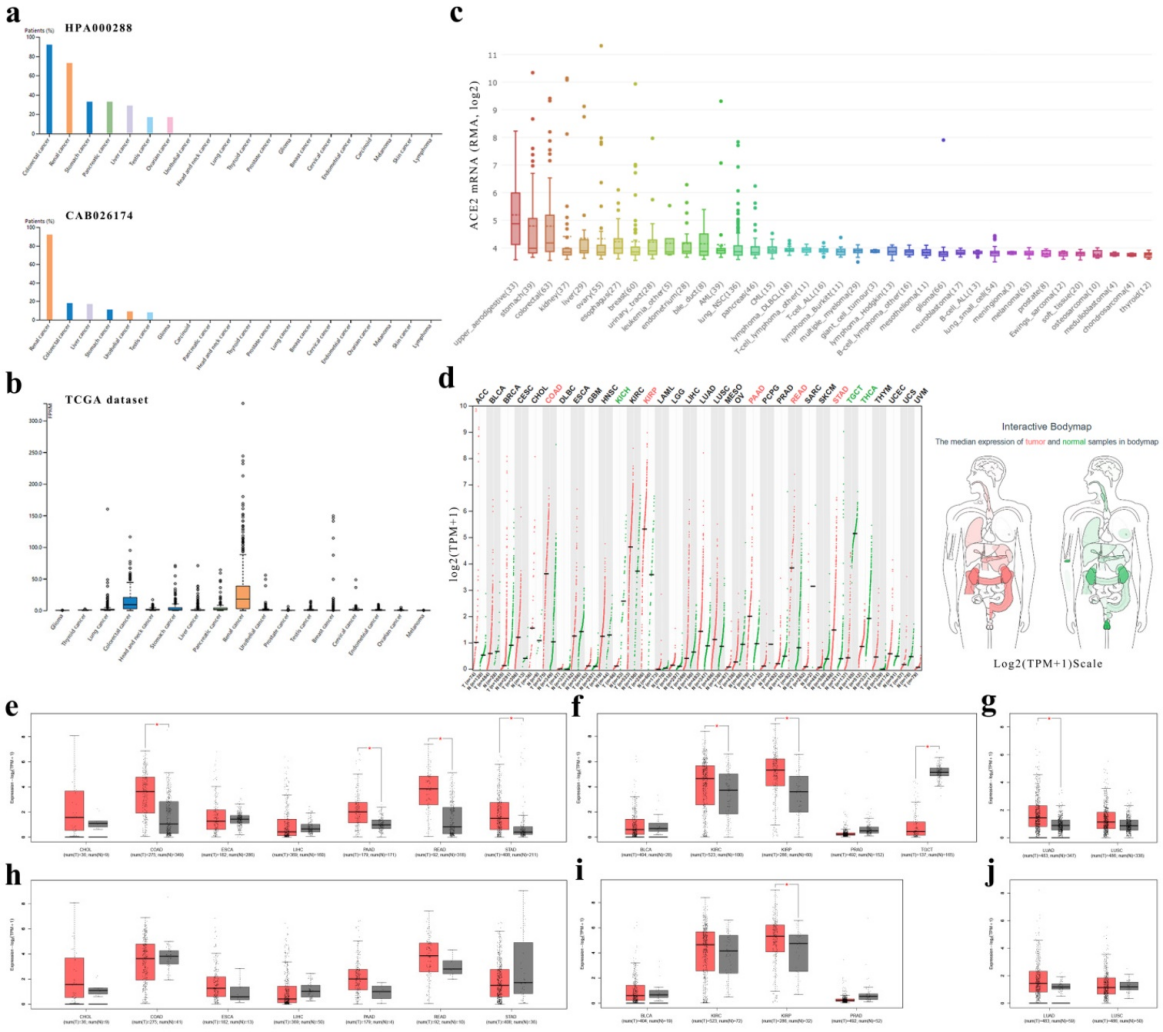
** ACE2 expression levels in human cancers.** (**a**) The protein expressions of ACE2 in cancer tissues, which were ranked by the expression level, as assessed by HPA000288 and CAB026174 antibodies. The y-axis represents the percent of patients (maximum 12 patients) with high or medium expression levels. The data were obtained from the pathology atlas. (**b**) Overview of RNA expressions in different cancers. The data were obtained from the TCGA dataset in the pathology atlas. (**c**) Box plot showing the mRNA expression levels of ACE2 in human cancer cell lines from the CCLE database. Within each box, the median is a solid line, while the mean is a dashed line. (**d**) Bar plot and interactive body map of ACE2 expression levels in cancer samples and matched normal samples through GEPIA (TCGA normal + GTEx normal); each dot represents a sample. (**e-j**) Boxplot showing ACE2 expressions in human cancer and matched normal tissue (TCGA normal + GTEx normal) (e-g) or in human cancer and adjacent normal tissue (TCGA) (h-j), as obtained using GEPIA. The y-axis represents transformed log2(TPM+1). The |log2FC| cutoff is 0.5 and the p-value cutoff is 0.05. The jitter size is 0.4 (red color: cancer samples; gray color: normal samples). RMA, Robust Multi-array Average; CCLE, Cancer Cell Line Encyclopedia; TPM, transcripts per million; TCGA, The Cancer Genome Atlas; GTEx, Genotype-Tissue Expression; GEPIA, Gene Expression Profiling Interactive Analysis; CHOL, cholangiocarcinoma; COAD, colon adenocarcinoma; ESCA, esophageal carcinoma; LIHC, liver hepatocellular carcinoma; PAAD, pancreatic adenocarcinoma; READ, rectum adenocarcinoma; STAD, stomach adenocarcinoma; BLCA, bladder urothelial carcinoma; KIRC, kidney renal clear cell carcinoma; KIRP, kidney renal papillary cell carcinoma; PRAD, prostate adenocarcinoma; TGCT, testicular germ cell tumor; LUAD, lung adenocarcinoma; LUSC, lung squamous cell carcinoma.

First, we found that expressions of ACE2 in cancers of the digestive, urinary or male reproductive tracts were generally higher than that in other cancers (Fig. 2a-c and Table S2).

Second, we analyzed the ACE2 mRNA expression profiles across multiple cancer types and matched normal tissues (from both TCGA and GTEx) in GEPIA (Fig. 2d-g) and found that colon adenocarcinoma (COAD), PAAD, rectum adenocarcinoma (READ) and stomach adenocarcinoma (STAD) expressed ACE2 at higher levels than matched normal tissues in the digestive tract; however, there was no statistical significance in cholangiocarcinoma (CHOL), esophageal carcinoma (ESCA) or LIHC (Fig. 2e). In the urinary and male reproductive tracts, ACE2 was expressed at a higher level in KIRC and kidney renal papillary cell carcinoma (KIRP) than in normal kidney, but at a lower level in testicular germ cell tumor (TGCT) than in normal testis; nevertheless, the difference in bladder urothelial carcinoma (BLCA) and prostate adenocarcinoma (PRAD) did not reach statistical significance (Fig. 2f). Additionally, for the respiratory tract, ACE2 expression in lung adenocarcinoma (LUAD) was higher than that in normal lung (Fig. 2g).

In GEPIA, it should be noted that the matched GTEx data for READ were from the healthy colon; and matched GTEx data for ESCA included gastroesophageal junction, mucosa and muscularis. Therefore, we excluded the data in GTEx and found no significant differences between cancer and adjacent normal tissues, except in KIRP, indicating a difference between normal data in TCGA and data in GTEx. In general, the expression level of ACE2 in healthy tissues was lower than that in adjacent normal tissues (Fig. 2e-j). Additionally, by using GEPIA, we found that early-stage tumors tend to express ACE2 at a higher level than advanced tumors in KIRC and KIRP. However, there was no statistically significant difference in the expression level of ACE2 among different stages of other cancer types (Fig. S2).

**Figure 3:**
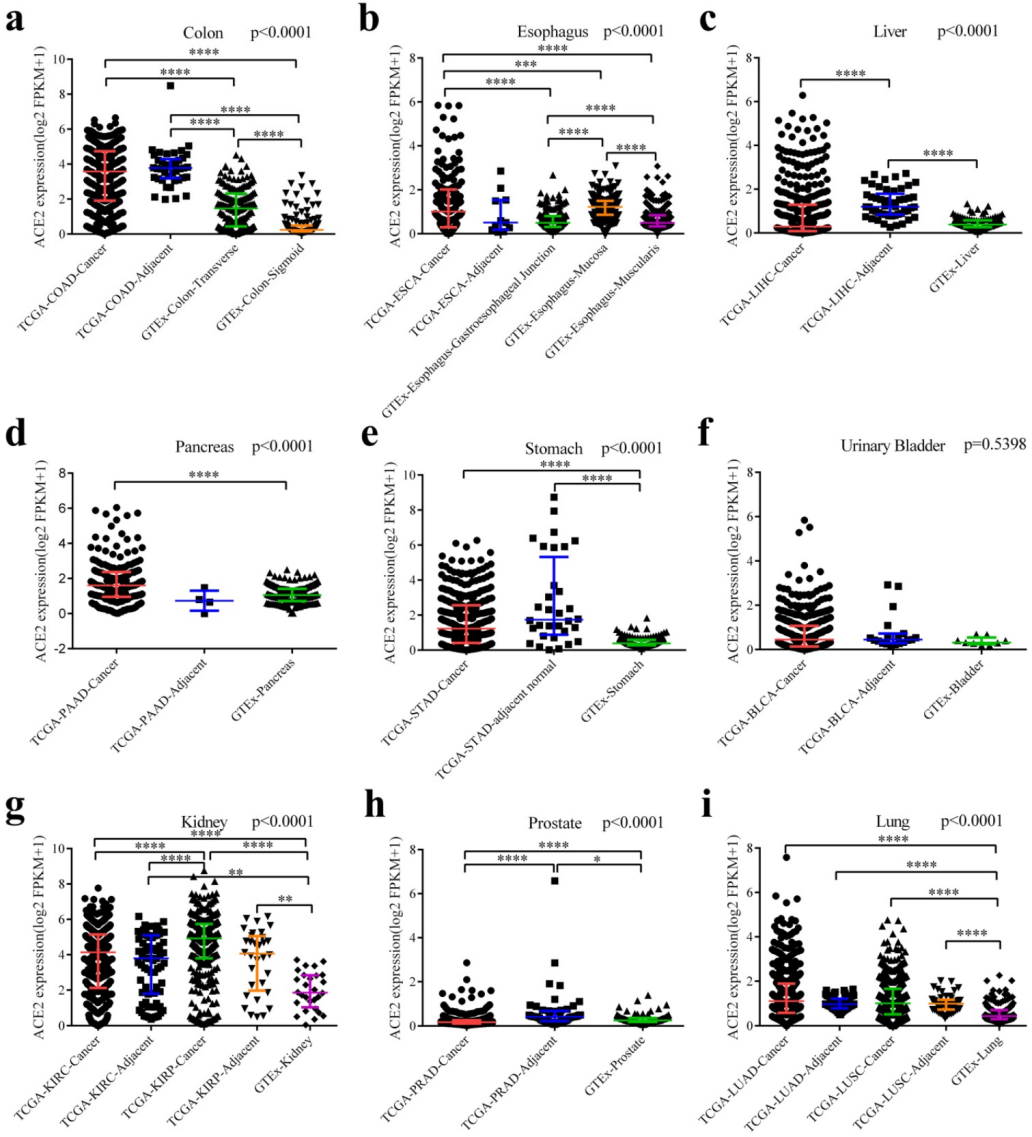
** Differential expressions of ACE2 among cancers, adjacent normal tissues and healthy tissues.** (**a**) Differential expressions among COAD, adjacent normal colon and healthy colon (including transverse and sigmoid colon). (**b**) Differential expressions among ESCA, adjacent normal esophagus and healthy esophagus (including gastroesophageal junction, mucosa and muscularis). (**c**) Differential expressions among LIHC, adjacent normal liver and healthy liver. (**d**) Differential expressions among PDAC, adjacent normal pancreas and healthy pancreas. (**e**) Differential expressions among STAD, adjacent normal stomach and healthy stomach. (**f**) Differential expressions among BLCA, adjacent normal bladder and healthy bladder. (**g**) Differential expressions among KIRC (together with adjacent normal kidney), KIRP (together with adjacent normal kidney) and healthy kidney. (**h**) Differential expressions among PRAD, adjacent normal prostate and healthy prostate. (**i**) Differential expressions among LUAD (together with adjacent normal lung), LUSC (together with adjacent normal lung) and healthy lung. Kruskal-Wallis tests were performed followed by pairwise post-hoc analysis. TCGA, The Cancer Genome Atlas; GTEx, Genotype-Tissue Expression; FPKM, number of fragments per kilobase of exon per million reads; COAD, colon adenocarcinoma; ESCA, esophageal carcinoma; LIHC, liver hepatocellular carcinoma; PAAD, pancreatic adenocarcinoma; STAD, stomach adenocarcinoma; BLCA, bladder urothelial carcinoma; KIRC, kidney renal clear cell carcinoma; KIRP, kidney renal papillary cell carcinoma; PRAD, prostate adenocarcinoma; LUAD, lung adenocarcinoma; LUSC, lung squamous cell carcinoma. **, P < 0.01; ***, P < 0.001; **** P < 0.0001.

Finally, we downloaded the original data and compared ACE2 expression among cancers (from TCGA), adjacent normal tissues (from TCGA) and healthy tissues (from GTEx). As generally shown in Fig. 3, adjacent normal tissues expressed ACE2 at a different manner than healthy tissues. ACE2 expression in adjacent normal colon was higher than in transverse or sigmoid colon, and these two sections of colon expressed ACE2 at a different level (Fig. 3a). In healthy esophagus, mucosa expressed higher level of ACE2 compared to muscularis (Fig. 3b). Intriguingly, ACE2 expression in adjacent normal liver was higher than both LIHC and healthy liver (Fig. 3c); and a same trend was observed in prostate (Fig. 3h). Both cancers and adjacent normal tissues expressed ACE2 at higher levels than healthy tissues in colon (Fig. 3a), stomach (Fig. 3e), kidney (Fig. 3g) and lung (Fig. 3i). Moreover, we have performed pairwise comparisons between cancers and adjacent normal tissues (Fig. S3), and the results were similar to that in Fig. 3.

### ACE2 expression correlated with metabolic and immune-related pathways across multiple cancer types

**Figure 4:**
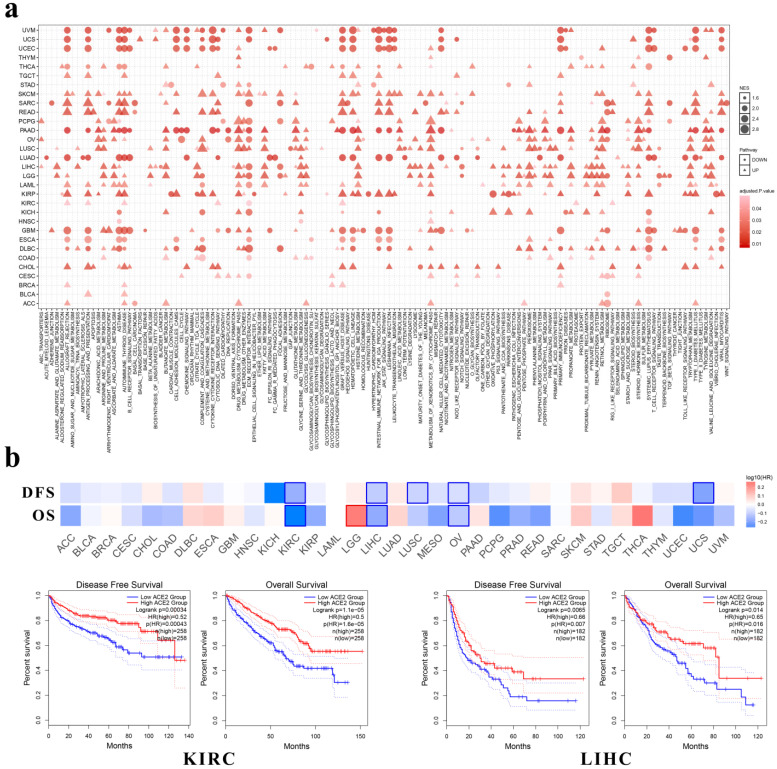
** Enriched pathways and prognostic value of ACE2 expression in multiple cancer types.** (**a**) KEGG pathway analysis was performed using GSEA. The x-axis represents different KEGG pathways (triangles: positively correlated pathways; circles: negatively correlated pathways). (**b**) Survival significance map of ACE2 showed the survival analysis results, including OS and DFS, across multiple cancer types through GEPIA (the red and blue blocks denote higher and lower risks, respectively; the rectangles with frames indicate significant unfavorable and favorable results). The lower panel shows the Kaplan-Meier plots of OS and DFS in KIRC and LIHC. Median ACE2 expression values were adopted as the cutoff to divide the patients into high-expression and low-expression groups. The log-rank test was used without P-value adjustment. KEGG, Kyoto Encyclopedia of Genes and Genomes; GSEA, gene set enrichment analysis; NES, normalized enrichment score; OS, overall survival; DFS, disease-free survival; HR, hazard ratio; KIRC, kidney renal clear cell carcinoma; LIHC, liver hepatocellular carcinoma. P < 0.05 was considered statistically significant.

Through GSEA we identified the enriched KEGG pathways by ACE2. As shown in Fig. 4a, across multiple cancer types, ACE2 expression correlated significantly with metabolic pathways, such as arginine and proline metabolism, ascorbate and aldarate metabolism, butanoate metabolism, drug metabolism-cytochrome P450, and peroxisome. In addition, ACE2 expression correlated significantly with immune-related pathways, such as allograft rejection, antigen processing and presentation, autoimmune thyroid disease, intestinal immune network for IgA production, and primary immunodeficiency.

Among these cancers, ACE2 expression correlated positively with immune-related pathways in CHOL, KIRP and READ, whereas it had negative correlations with immune-related pathways in ESCA, PAAD and TGCT. Additionally, in LUAD and lung squamous cell carcinoma (LUSC), we found a negative correlation between ACE2 expression and the olfactory or taste transduction pathways.

Moreover, we investigated the clinical relevance of ACE2 expression through GEPIA and found that the expression of ACE2 was associated with better OS and DFS in KIRC, LIHC (Fig. 4b) and ovarian serous cystadenocarcinoma (OV) patients. In addition, ACE2 expression correlated with better DFS in LUSC and uterine carcinosarcoma (UCS). However, its expression correlated with worse OS in brain lower grade glioma (LGG).

### ACE2 expression correlated with immune cell infiltration in both cancer and normal tissues

Based on the above results, the correlation between ACE2 expression and immune infiltration levels was further investigated.

First, as is shown in the heatmap (Fig. S4a), ACE2 was positively and significantly associated with the infiltration of at least two types of immune cells in BLCA, breast invasive carcinoma (BRCA), KIRC, OV, PAAD, PRAD, READ, primary skin cutaneous melanoma (SKCM), thyroid carcinoma (THCA) and uterine corpus endometrial carcinoma (UCEC). However, there were generally negative correlations with immune cell infiltration in COAD, LIHC, LUSC and thymoma (THYM).

**Figure 5:**
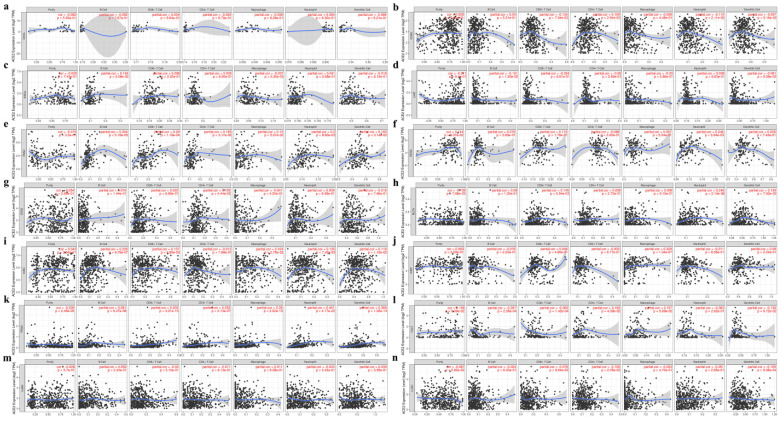
** Correlation between ACE2 expression and tumor purity or immune infiltration levels in multiple cancer types through TIMER.** (**a-g**) Scatter plots present the correlations between ACE2 expression and tumor purity or immune cell infiltration in CHOL, COAD, ESCA, LIHC, PAAD, READ and STAD (cancers of the digestive tracts). (**h-l**) Scatter plots present the correlations between ACE2 expression and tumor purity or immune cell infiltration in BLCA, KIRC, KIRP, PRAD and TGCT (cancers of urinary and male reproductive tracts). (**m and n**) Scatter plots present the correlations between ACE2 expression and tumor purity or immune cell infiltration in lung cancers, including LUAD and LUSC. The x-axis represents the infiltration level. Partial_Cor, purity-adjusted partial Spearman's correlation; CHOL, cholangiocarcinoma; COAD, colon adenocarcinoma; ESCA, esophageal carcinoma; LIHC, liver hepatocellular carcinoma; PAAD, pancreatic adenocarcinoma; READ, rectum adenocarcinoma; STAD, stomach adenocarcinoma; BLCA, bladder urothelial Carcinoma; KIRC, kidney renal clear cell carcinoma; KIRP, kidney renal papillary cell carcinoma; PRAD, prostate adenocarcinoma; TGCT, testicular germ cell tumor; LUAD, lung adenocarcinoma; LUSC, lung squamous cell carcinoma. P < 0.05 was considered statistically significant.

Second, we explored the correlation coefficients in cancers of the digestive, urinary, male reproductive and respiratory tracts. No significant correlation was found in CHOL (Fig. 5a), whereas in other cancers ACE2 expression had a significant correlation with infiltrating immune cells (Fig. 5b-l). Additionally, for lung cancers, we found no significant correlation in LUAD (Fig. 5m and 5n).

Finally, by using TIMER and GEPIA, we investigated the correlations between ACE2 and related markers of various immune cells, including CD8+ T cell, T cell (general), B cell, monocyte, tumor-associated macrophage (TAM), M1/M2 macrophage, neutrophil, natural killer (NK) cell, dendritic cell (DC), T-helper 1 (Th1) cell, T-helper 2 (Th2) cell, follicular helper T (Tfh) cell, T-helper 17 (Th17) cell, regulatory T cell (Treg) and exhausted T cell. Similar to the results described above, after adjustment for purity, a heatmap in TIMER (Fig. S4b) showed that ACE2 was significantly associated with most immune marker sets in various types of human cancers. We further analyzed the correlation between ACE2 expression and the above immune markers in cancers, including KIRC, PAAD and PRAD, through GEPIA (Table 2), the results were similar to those in TIMER; meanwhile, we found significant correlations in normal tissues (from both TCGA and GTEx).

Therefore, we extended this analysis to other normal tissues (Table 3). Similar to the results in cancer tissues, the correlations were also tissue type-dependent, with high correlation coefficients in the normal stomach and colon, which were even higher than those in corresponding cancer tissues. Whereas, there was almost no statistically significant correlation in bladder.

## Discussion

The worldwide outbreak of COVID-19 has been a severe challenge for public health and the economy, exploring the susceptibility to COVID-19 and its severity in the population is of paramount importance.

In this study, we found that glandular cells in the digestive, urinary and male reproductive tracts expressed ACE2 at high levels (Fig. 1), which is generally consistent with the findings of previous studies 4, 5. In contrast, blood cells expressed almost no ACE2 (Fig. S1), although others demonstrated that classical monocytes (CD14^++^CD16^-^) expressed ACE2 at a relatively high level 28. Moreover, by analyzing single-cell RNA-seq data through HCL, we found that ACE2-expressing cells were mainly from the fetal intestine, fetal adrenal gland, fetal kidney, adult intestine, adult kidney and adult gallbladder (Table 1). Others have detected SARS-CoV-2 IgM antibodies in neonatal sera samples that were collected at birth 29. These results raised the possibility of fecal-oral and maternal-fetal transmission of SARS-CoV-2, which needs to be further validated 30. In addition, SARS-CoV-2 may infect other tissues besides the lung or persist in extrapulmonary tissues even after the cure of pneumonia 31. Other studies reported that the characteristics of pediatric SARS-CoV-2 infection differed from those of adult patients, with pediatric patients having milder respiratory symptoms but demonstrating persistently positive rectal swabs, even after the nasopharyngeal testing was negative 31, 32, which could be ascribed to the high expression of ACE2 in the gastrointestinal tracts in children.

Cancer is a disorder with immune dysfunction 11, and cancer patients are more susceptible to infections. In this study, we found that ACE2 was expressed at a high level in cancers of the digestive and urinary tracts; and ACE2 expressions in COAD, PAAD, STAD, KIRC, KIRP and LUAD were higher than that in matched normal tissues (including TCGA and GTEx data; Fig. 2). The results were generally same as other study 33. In addition, others have indicated that adjacent normal tissues presented a unique intermediate state between healthy and tumor 34; in this study, we found that the ACE2 expression level in adjacent normal tissues was generally higher than that in healthy tissues (Fig. 3). For example, other research has found that adjacent mucous membrane from COAD expressed ACE2 at a higher level than normal mucous membrane from healthy individuals 35. Moreover, sigmoid and transverse colons expressed ACE2 at different levels. These abovementioned results indicated that it was inappropriate to combine adjacent normal tissues (TCGA) and healthy tissues (GTEx) for comparison of ACE2 expression between tumor and normal tissues; and that cancer patients could be more susceptible to SARS-CoV-2 infection 14, 15, possibly through respiratory or digestive routes.

GSEA demonstrated that ACE2 was widely associated with immune-related signaling pathways (Fig. 4a). On the one hand, SARS-CoV-2 has been reported to be associated with lymphopenia (especially in severe cases 6, 8) and the infiltration of a large number of immune cells at infection sites 3, 10. On the other hand, cancer patients harbor dysfunctional immune cells in both the peripheral blood and inflammatory TME 11. Nevertheless, the correlation among SARS-CoV-2, cancer and immune infiltration has not been fully described. First, we found no significant correlation with tumor purity, except in BLCA and PRAD (Fig. 5), which could be explained by ACE2 expression in stromal cells, such as myofibroblasts and vascular cells 5. Intriguingly, we found that ACE2 expression correlated significantly with different immune cell infiltration in various tumor tissues, especially in PAAD, KIRC and PRAD, but not in CHOL or LUAD (Fig. 5) 36.

For cancer patients, once infection occurred either in the lung or the cancer, immune cells, including T cell, monocyte and macrophage 3, 9, were mobilized rapidly into the sites of infection to clear SARS-CoV-2, which could partially explain the lymphopenia and decreased monocytes in blood 8. Until now, conclusions about the severity of COVID-19 in cancer patients, when compared with the general population, have been controversial in different studies with small sample sizes 14, 15. Considering that immune cells in cancer patients are generally blunt, we postulated that COVID-19 in cancer patients would be more prolonged than in the general population.

Intriguingly, we found that ACE2 expression correlated significantly with the gene markers of various immune cells in normal tissues (Table 2 and Table 3). The immune infiltration levels among healthy tissues, adjacent normal tissues and cancers were different 35, 37, 38. Inflammatory response-related pathways were enriched in the adjacent normal tissues in most tissue types 34, and cancers such as STAD, LIHC, COAD and PAAD correlated significantly with chronic inflammation 39. Based on above results (Fig. 4, Fig. 5, Table 2 and Table 3), this immune infiltration pattern could partially explain the results that ACE2 expression in cancers or adjacent normal tissues was generally higher than that in healthy tissues (Fig. 3). As for differential ACE2 expression between cancers and adjacent normal tissues (Fig. 3 and Fig. S3), LIHC expressed ACE2 at a lower level than adjacent normal liver (Fig. 3c and Fig. S3c) 17, which may be related to the lower infiltration of immune cells in the cancer tissue, when compared with adjacent normal liver tissue 40. Similarly, PARD and PAAD were classified as immunologically “cold” with low immune cell infiltration 41, 42, which may contribute to the low cancerous ACE2 expression (Fig. 3h and Fig. S3h) 18; however, we found no significant difference in ACE2 expression between PAAD and adjacent normal pancreas (Fig. 3d and Fig. S3d), the discrepancy may be attributable to the small sample size of adjacent normal pancreas in our study (n = 4). ACE2 expression correlated with almost no gene markers of immune cells in bladder (Table 3), and there were no significant differences among BLCA, adjacent and normal bladders (Fig. 3). Therefore, we supposed that inflammation would increase ACE2 expression 43, 44, thus rendering people more susceptible to SARS-CoV-2. Similarly, others suggested that patients with inflammatory bowel disease (IBD) might be at an increased risk of SARS-CoV-2 infection 45.

It should be noted that other molecules have also been identified for host cell entry 2. In addition, although others reported that ACE2 protein and mRNA levels were strongly correlated 44, we analyzed only the mRNA expression of ACE2, but not the cell-surface expression. Since our data were from public databases and there were limited biopsy samples in other studies, whether SARS-CoV-2 could infect other tissues, such as cancers, merits further study. The predominant immune cells varied across different cancer types, therefore, the correlation between ACE2 expression and immune infiltration needs experimental evidences.

## Conclusions

In this study, we provided evidence for the tissue tropism of SARS-CoV-2 in both the adult and the fetus, although no reliable evidence is available to support the intrauterine infection caused by vertical transmission. Moreover, ACE2 expressions in COAD, ESCA, PAAD, STAD, KIRC, KIRP, LUAD and LUSC were higher than that in matched healthy tissues, providing a molecular rationale explaining why cancer patients could be more susceptible to SARS-CoV-2. In addition, we revealed a link between ACE2 expression and immune cell infiltration and indicated that adjacent normal tissues generally expressed ACE2 at higher levels than healthy tissues. Therefore, more intensive surveillance should be considered for respiratory and fecal-oral transmission in the patients with inflammation or cancers, and the immune response of cancer patients. Further investigation on the susceptibility to and severity of COVID-19 in cancer patients is urgently needed.

## Supplementary Material

Supplementary figures and tables.

## Figures and Tables

**Table 1:** ACE2 expression by single cell through the human cell landscape

Cluster	Cell type	Expression level	Tissue sources	Cell number	
				Total	Contribution
15	Fetal enterocyte	5.53	Adult ileum	3367	26
			Adult jejunum	5549	22
			Fetal adrenal gland	14416	1563
			Fetal intestine	23516	9935
			Fetal kidney	22662	10
			Fetal stomach	7953	166
16	Hepatocyte/ Endodermal cell	9.08	Adult jejunum	5549	10
			Fetal adrenal gland	14416	1001
			Fetal intestine	23516	10232
			Fetal stomach	7953	15
36	Fibroblast	1.69	Adult gallbladder	14800	6797
			Adult stomach	14624	47
39	Enterocyte	7.39	Adult duodenum	4681	1504
			Adult ileum	3367	1204
			Adult jejunum	5549	3852
			Adult transverse colon	16994	13
			Fetal intestine	23516	11
59	Epithelial cell	6.62	Adult kidney	22968	4396
81	Epithelial cell	2.42	Adult ascending colon	2026	12
			Adult rectum	5718	178
			Adult sigmoid colon	3281	102
			Adult transverse colon	16994	1801
83	Proximal tubule progenitor	6.48	Fetal adrenal gland	14416	19
			Fetal kidney	22662	1970
			Fetal male gonad	13211	21
91	Goblet cell	6.71	Adult gallbladder	14800	1202

Expression level, log2(clusterMean_TPM+1); Total, the number of cells isolated from the tissue; Contribution, the number of cells belonging to the cluster; TPM, transcripts per million.

**Table 2:** The correlation analysis between ACE2 and gene markers of immune cells in cancers and match normal tissues through GEPIA

Description	Gene markers	KIRC				PAAD				PRAD			
		Tumor		Normal		Tumor		Normal		Tumor		Normal	
		Cor	*P*	Cor	*P*	Cor	*P*	Cor	*P*	Cor	*P*	Cor	*P*
CD8^+^ T cell	CD8A	0.13	**	0.25	**	0.36	****	0.16	*	0.41	****	0.32	****
	CD8B	0.14	**	0.38	****	0.23	**	0.091	0.24	0.25	****	0.088	0.28
T cell (general)	CD3D	0.053	0.23	0.25	**	0.31	****	0.11	0.14	0.33	****	0.16	*
	CD3E	0.086	0.05	0.37	****	0.36	****	0.097	0.21	0.38	****	0.22	**
	CD2	0.12	**	0.34	****	0.37	****	0.13	0.093	0.39	****	0.22	**
B cell	CD19	-0.15	***	0.075	0.35	0.15	0.052	0.17	*	0.24	****	-0.018	0.82
	CD79A	-0.11	*	0.4	****	0.17	*	0.16	*	0.25	****	0.086	0.29
Monocyte	CD86	0.17	***	0.39	****	0.15	*	0.26	***	0.42	****	0.24	**
	CD115 (CSF1R)	0.12	**	0.43	****	0.24	**	0.2	**	0.41	****	0.19	*
TAM	CCL2	0.29	****	-0.16	0.05	0.21	**	0.23	**	0.36	****	0.12	0.15
	CD68	0.22	****	0.72	****	0.2	**	0.21	**	0.37	****	0.27	***
	IL10	0.047	0.29	0.1	0.2	0.11	0.13	0.37	****	0.3	****	0.32	****
M1 Macrophage	INOS (NOS2)	0.2	****	0.21	**	0.1	0.18	0.082	0.29	0.37	****	0.14	0.091
	IRF5	0.28	***	-0.55	****	0.081	0.28	0.19	*	0.34	****	0.46	****
	COX2 (PTGS2)	-0.19	****	-0.21	**	-0.082	0.27	0.059	0.45	0.35	****	0.29	***
M2 Macrophage	CD163	-0.055	0.21	0.23	**	0.18	*	0.4	****	0.26	****	0.29	***
	VSIG4	-0.022	0.62	0.3	***	0.15	*	0.38	****	0.36	****	0.31	***
	MS4A4A	0.048	0.27	0.43	****	0.19	*	0.34	****	0.3	****	0.3	***
Neutrophil	CD66b (CEACAM8)	0.05	0.26	-0.17	*	0.22	**	0.016	0.83	0.095	*	0.5	****
	CD11b (ITGAM)	0.17	****	0.38	****	0.17	*	0.27	***	0.42	****	0.24	**
	CCR7	0.019	0.67	0.21	**	0.27	***	0.38	****	0.34	****	0.5	****
Natural killer cell	KIR2DL1	0.19	****	0.25	**	0.089	0.24	-0.12	0.1	0.21	****	0.036	0.66
	KIR2DL3	0.17	***	0.28	***	0.093	0.22	0.068	0.37	0.1	*	0.17	*
	KIR2DL4	0.066	0.13	0.13	*	0.055	0.46	0.13	0.1	0.16	****	0.096	0.24
	KIR3DL1	0.21	****	0.27	***	0.001	1	0.096	0.21	0.093	*	-0.003	0.97
	KIR3DL2	0.17	****	0.2	*	0.24	***	-0.069	0.37	0.14	**	0.17	*
	KIR3DL3	-0.078	0.07	0.055	0.5	0.11	0.14	-0.053	0.49	0.092	*	0.01	0.9
	KIR2DS4	0.11	*	0.13	0.11	-0.017	0.82	0.012	0.87	0.15	***	0.17	*
Dendritic cell	HLA-DPB1	0.25	****	0.14	0.082	0.28	***	0.12	0.13	0.38	****	-0.023	0.78
	HLA-DQB1	0.16	***	-0.031	0.7	0.16	*	-0.052	0.5	0.26	****	0.074	0.37
	HLA-DRA	0.27	****	0.13	0.095	0.27	***	0.11	0.14	0.43	****	0.11	0.16
	HLA-DPA1	0.29	****	0.16	*	0.26	***	0.053	0.5	0.4	****	-0.021	0.8
	BDCA-1 (CD1C)	0.21	****	0.41	****	0.42	****	0.076	0.32	0.4	****	0.039	0.63
	BDCA-4 (NRP1)	0.25	****	0.53	****	0.21	**	0.19	*	0.26	****	0.079	0.33
	CD11c (ITGAX)	0.041	0.35	0.17	*	0.12	0.099	0.15	0.053	0.36	****	0.11	0.19
Th1	T-bet (TBX21)	0.11	*	0.26	***	0.3	****	0.14	0.077	0.37	****	0.26	**
	STAT4	0.018	0.68	0.064	0.43	0.26	***	0.18	*	0.42	****	0.14	0.089
	STAT1	0.23	****	-0.14	0.086	0.18	*	0.12	0.12	0.36	****	0.17	*
	IFN-g (IFNG)	0.08	0.07	0.006	0.94	0.084	0.26	-0.013	0.86	0.27	****	0.069	0.4
	TNF-a (TNF)	0.073	0.09	-0.18	*	0.13	0.076	0.26	***	0.35	****	0.28	***
Th2	GATA3	-0.17	***	-0.35	****	-0.09	0.23	0.24	**	0.48	****	0.34	****
	STAT6	0.26	****	-0.21	**	0.32	****	0.17	*	0.27	****	0.19	*
	STAT5A	0.2	****	0.23	**	0.24	**	0.27	***	0.44	****	0.31	****
	IL13	-0.041	0.35	-0.087	0.28	-0.07	0.35	0.06	0.43	0.13	**	0.11	0.17
Tfh	BCL6	0.01	0.82	-0.52	****	0.17	*	0.17	*	0.29	****	0.26	**
	IL21	-0.032	0.47	0.18	*	0.099	0.19	-0.028	0.72	0.18	****	-0.018	0.82
Th17	STAT3	0.22	****	-0.19	*	0.26	***	0.13	0.08	0.42	****	0.24	**
	IL17A	-0.015	0.73	-0.042	0.6	0.16	*	0.051	0.51	0.23	****	0.23	**
Treg	FOXP3	-0.13	**	-0.08	0.32	0.2	**	0.18	*	0.29	****	0.22	**
	CCR8	0.06	0.17	0.42	****	0.23	**	0.008	0.91	0.27	****	0.15	0.063
	STAT5B	0.41	****	0.25	**	0.22	**	0.23	**	0.33	****	0.15	0.06
	TGFb (TGFB1)	-0.27	****	-0.43	****	-0.11	0.15	0.096	0.21	0.37	****	0.19	*
T cell exhaustion	PD-1 (PDCD1)	0.062	0.16	0.34	****	0.26	***	0.14	0.067	0.31	****	0.22	**
	CTLA4	0.033	0.45	0.13	0.096	0.2	**	0.14	0.067	0.26	****	0.23	**
	LAG3	0.036	0.41	-0.53	****	0.095	0.21	0.13	0.098	0.24	****	0.097	0.23
	TIM-3 (HAVCR2)	0.37	****	0.82	****	0.15	0.051	0.24	**	0.42	****	0.3	***
	GZMB	-0.0091	0.84	0.29	***	0.15	0.05	-0.01	0.9	0.39	****	0.21	**

KIRC, kidney renal clear cell carcinoma; PAAD, pancreatic adenocarcinoma; PRAD, prostate adenocarcinoma; Cor, R value of Spearman's correlation; TAM, tumor-associated macrophage; Th1, T-helper 1; Th2, T-helper 2; Tfh, follicular helper T; Th17, T-helper 17; Treg, regulatory T cell. *, p < 0.05; **, p < 0.01; ***, p < 0.001; ****, p < 0.0001.

**Table 3:** The correlation analysis between ACE2 and gene markers of immune cells in normal tissues through GEPIA

Description	Gene markers	Lung		Esophagus		Stomach		Colon		Liver		Bladder		Testis	
		Cor	*P*	Cor	*P*	Cor	*P*	Cor	*P*	Cor	*P*	Cor	*P*	Cor	*P*
CD8^+^ T cell	CD8A	-0.16	**	0.26	****	0.27	****	0.68	****	0.23	**	0.15	0.44	0.033	0.68
	CD8B	-0.12	*	0.23	****	0.26	***	0.67	****	0.23	**	0.19	0.34	0.015	0.84
T cell (general)	CD3D	-0.14	**	0.34	****	0.33	****	0.68	****	0.25	**	0.14	0.48	0.056	0.48
	CD3E	-0.014	0.78	0.34	****	0.37	****	0.72	****	0.31	****	0.14	0.48	-0.28	***
	CD2	0.004	0.93	0.37	****	0.36	****	0.69	****	0.3	***	0.13	0.5	-0.57	****
B cell	CD19	-0.21	****	0.018	0.76	0.3	****	0.51	****	-0.014	0.86	-0.31	0.11	-0.5	****
	CD79A	0.043	0.4	0.18	**	0.32	****	0.74	****	0.26	***	-0.057	0.77	0.017	0.82
Monocyte	CD86	0.2	****	0.21	***	0.58	****	0.44	****	0.17	*	0.039	0.84	-0.3	***
	CD115 (CSF1R)	0.12	*	0.16	**	0.42	****	0.13	*	0.095	0.23	-0.022	0.91	0.24	**
TAM	CCL2	-0.2	****	-0.26	****	0.16	*	-0.49	****	0.012	0.88	-0.16	0.43	0.32	****
	CD68	0.45	****	0.48	****	0.66	****	0.62	****	0.066	0.41	0.48	**	0.22	**
	IL10	0.088	0.08	0.28	****	0.36	****	0.11	*	-0.083	0.3	0.14	0.47	-0.47	****
M1 Macrophage	INOS (NOS2)	-0.24	****	0.055	0.36	0.31	****	0.73	****	0.35	****	-0.12	0.54	0.23	**
	IRF5	-0.1	*	0.059	0.32	0.48	****	0.46	****	-0.12	0.13	0.41	*	0.28	***
	COX2 (PTGS2)	-0.18	***	-0.39	****	0.11	0.11	-0.6	****	0.016	0.84	-0.05	0.8	0.19	*
M2 Macrophage	CD163	0.32	****	0.3	****	0.13	0.06	0.039	0.47	-0.002	0.98	-0.16	0.4	0.28	***
	VSIG4	0.4	****	0.34	****	0.34	****	0.18	***	0.024	0.77	-0.17	0.37	0.28	***
	MS4A4A	0.33	****	0.29	****	0.33	****	0.18	***	0.078	0.32	-0.19	0.32	0.3	****
Neutrophil	CD66b (CEACAM8)	-0.11	*	-0.13	*	0.086	0.21	0.66	****	-0.056	0.48	0.15	0.44	-0.15	0.05
	CD11b (ITGAM)	-0.048	0.34	0.13	*	0.27	****	-0.33	****	-0.2	**	-0.16	0.41	0.3	****
	CCR7	0.026	0.6	0.091	0.12	0.51	****	0.29	****	-0.19	*	0.24	0.21	0.22	**
Natural killer cell	KIR2DL1	-0.24	****	-0.07	0.24	0.16	*	0.11	*	0.034	0.67	0.31	0.11	0.015	0.85
	KIR2DL3	-0.21	****	-0.021	0.72	0.32	****	0.3	****	0.07	0.38	0.35	0.07	0.16	*
	KIR2DL4	-0.16	**	0.014	0.82	0.47	****	0.76	****	-0.1	0.2	0.54	**	-0.081	0.3
	KIR3DL1	-0.11	*	-0.12	0.05	0.2	**	0.3	****	0.013	0.87	0.23	0.24	-0.001	0.99
	KIR3DL2	-0.05	0.32	0.05	0.4	0.26	***	0.52	****	0.12	0.13	0.37	0.05	0.014	0.86
	KIR3DL3	-0.096	0.06	0.12	0.05	0.13	0.06	0.32	****	0.089	0.26	-0.12	0.56	0.059	0.45
	KIR2DS4	-0.2	****	-0.11	0.06	0.21	**	0.23	****	0.11	0.18	0.21	0.29	-0.15	0.06
Dendritic cell	HLA-DPB1	0.2	****	0.034	0.57	0.59	****	0.25	****	0.37	****	0.077	0.7	0.41	****
	HLA-DQB1	-0.016	0.75	-0.1	0.08	0.39	****	0.16	**	0.23	**	0.091	0.64	-0.056	0.48
	HLA-DRA	0.24	****	0.15	*	0.63	****	0.35	****	0.36	****	0.12	0.55	0.34	****
	HLA-DPA1	0.23	****	0.094	0.11	0.58	****	0.29	****	0.32	****	0.12	0.56	0.29	***
	BDCA-1 (CD1C)	0.099	*	0.26	****	0.43	****	0.064	0.23	0.32	****	0.09	0.65	-0.17	*
	BDCA-4 (NRP1)	-0.15	**	-0.23	***	0.26	***	-0.55	****	-0.06	0.45	-0.15	0.46	0.4	****
	CD11c (ITGAX)	-0.43	****	-0.15	*	0.22	**	-0.19	***	-0.28	***	0.019	0.92	0.23	**
Th1	T-bet (TBX21)	-0.4	****	0.089	0.13	0.17	*	0.51	****	0.13	0.1	0.28	0.15	0.22	**
	STAT4	-0.49	****	0.004	0.95	0.16	*	0.3	****	-0.13	0.11	0.3	0.12	-0.51	****
	STAT1	0.039	0.43	0.18	**	0.51	****	-0.075	0.16	0.14	0.075	0.012	0.95	0.4	****
	IFN-g (IFNG)	-0.37	****	0.001	0.98	0.21	**	0.22	****	0.24	**	0.27	0.16	0.015	0.85
	TNF-a (TNF)	-0.23	****	0.21	***	0.38	****	0.26	****	0.077	0.33	-0.074	0.71	0.15	0.06
Th2	GATA3	-0.21	****	0.24	****	0.5	****	0.51	****	0.19	*	0.32	0.1	0.19	*
	STAT6	-0.29	****	-0.13	*	0.28	****	-0.28	****	-0.3	****	0.21	0.27	0.4	****
	STAT5A	-0.31	****	-0.17	**	0.28	****	-0.55	****	-0.28	***	-0.38	*	0.35	****
	IL13	-0.11	*	-0.13	*	-0.032	0.64	-0.26	****	0.18	*	-0.087	0.66	-0.56	****
Tfh	BCL6	-0.26	****	0.07	0.24	0.043	0.54	-0.57	****	-0.43	****	-0.11	0.58	0.2	*
	IL21	0.041	0.42	0.19	**	0.28	****	0.35	****	0.15	0.06	-0.088	0.66	-0.44	****
Th17	STAT3	-0.11	*	0.14	*	0.28	****	-0.34	****	-0.43	****	-0.1	0.6	0.52	****
	IL17A	-0.11	*	0.2	***	0.43	****	0.44	****	-0.015	0.85	-0.028	0.89	-0.042	0.6
Treg	FOXP3	0.13	*	0.36	****	0.48	****	0.63	****	0.46	****	0.24	0.23	0.18	*
	CCR8	0.044	0.38	0.22	***	0.44	****	0.47	****	0.067	0.4	0.31	0.11	-0.037	0.64
	STAT5B	-0.41	****	-0.21	***	0.089	0.2	-0.7	****	-0.3	****	-0.35	0.06	-0.033	0.67
	TGFb (TGFB1)	-0.41	****	0.032	0.59	0.42	****	-0.62	****	0.022	0.78	-0.064	0.75	0.48	****
T cell exhaustion	PD-1 (PDCD1)	-0.2	****	0.25	****	0.29	****	0.56	****	0.13	0.09	0.029	0.88	0.13	0.11
	CTLA4	-0.29	****	0.25	****	0.34	****	0.35	****	0.22	**	0.19	0.33	0.29	***
	LAG3	-0.39	****	-0.14	*	0.41	****	-0.34	****	0.2	**	0.011	0.96	0.33	****
	TIM-3 (HAVCR2)	0.008	0.87	0.2	***	0.5	****	0.32	****	0.007	0.93	0.045	0.82	-0.27	***
	GZMB	-0.24	****	0.03	0.61	0.4	****	0.51	****	0.1	0.2	0.2	0.3	0.1	0.2

Cor, R value of Spearman's correlation; TAM, tumor-associated macrophage; Th1, T-helper 1; Th2, T-helper 2; Tfh, follicular helper T; Th17, T-helper 17; Treg, regulatory T cell. *, p < 0.05; **, p < 0.01; ***, p < 0.001; ****, p < 0.0001.

## Abbreviations

ACE2: angiotensin-converting enzyme 2
BLCA: bladder urothelial carcinoma
BRCA: breast invasive carcinoma
CCLE: Cancer Cell Line Encyclopedia
CHOL: cholangiocarcinoma
COAD: colon adenocarcinoma
COVID-19: coronavirus disease 2019
DC: dendritic cell
DFS: disease-free survival
ESCA: esophageal carcinoma
FPKM: number of fragments per kilobase of exon per million reads
GEPIA: Gene Expression Profiling Interactive Analysis
GSEA: gene set enrichment analysis
GTEx: Genotype-Tissue Expression
HCL: human cell landscape
HPA: Human Protein Atlas
HR: hazard ratio
IBD: inflammatory bowel disease
KEGG: Kyoto Encyclopedia of Genes and Genomes
KIRC: kidney renal clear cell carcinoma
KIRP: kidney renal papillary cell carcinoma
LGG: brain lower grade glioma
LIHC: liver hepatocellular carcinoma
LUAD: lung adenocarcinoma
LUSC: lung squamous cell carcinoma
NES: normalized enrichment score
NK: natural killer
NX: consensus normalized expression
OS: overall survival
OV: ovarian serous cystadenocarcinoma
PAAD: pancreatic adenocarcinoma
Partial_Cor: purity-adjusted partial Spearman's correlation
PBMC: peripheral blood mononuclear cells
PRAD: prostate adenocarcinoma
pTPM: protein-coding transcripts per million
READ: rectum adenocarcinoma
RMA: Robust Multi‑array Average
SARS-CoV-2: severe acute respiratory syndrome coronavirus 2
SKCM: skin cutaneous melanoma
STAD: stomach adenocarcinoma
TAM: tumor associated macrophage
TCGA: The Cancer Genome Atlas
Tfh: follicular helper T
TGCT: testicular germ cell tumor
Th1: T-helper 1
Th2: T-helper 2
Th17: T-helper 17
THCA: thyroid carcinoma
THYM: thymoma
TIMER: Tumor Immune Estimation Resource
TME: tumor microenvironment
TPM: transcripts per million
Treg: regulatory T cell
UCEC: uterine corpus endometrial carcinoma
UCS: uterine carcinosarcoma
WHO: World Health Organization
