# Supplementary material for: Bioinformatic Analysis of Correlation between Immune Infiltration and COVID-19 in Cancer Patients
Source: Int J Biol Sci. 2020 Jul 6;16(13):2464–76. doi: 10.7150/ijbs.48639 (PMC7378636; doi:10.7150/ijbs.48639)
Supplement: Supplementary file 1 — Supplementary figures and tables. [file ijbsv16p2464s1.pdf]

Supplementary materials

Fig. S1. The mRNA expression of ACE2 in human peripheral blood cells.

(a) Consensus mRNA expression levels of ACE2 in 18 blood cell types and total PBMC obtained from HPA. (b) The mRNA expression levels of ACE2 in 18 blood cell types and total PBMC, RNA-seq data were from HPA. (c) The mRNA expression levels of ACE2 in 29 blood cell types and total PBMC, the data were obtained from Monoco publication. (d) The mRNA expression levels of ACE2 in 15 blood cell types, the data were obtained from Schmiedel publication. HPA, Human Protein Atlas; NX, consensus normalized expression; NK, natural killer; PBMC, peripheral blood mononuclear cells; pTPM, protein-coding transcripts per million; TPM, transcript per million.

Fig. S2. Correlations between ACE2 expression and tumor stages in multiple cancer types through GEPIA.

(a-g) Violin plots showing ACE2 expressions at different stages of CHOL, COAD, ESCA, LIHC, PAAD, READ and STAD (cancers of the digestive tracts). (h-k) Violin plots showing ACE2 expression at different stages of BLCA, KIRC, KIRP and TGCT (cancers of urinary and male reproductive tracts). (l and m) Violin plots showing ACE2 expression at different stages of lung cancers, including LUAD and LUSC. CHOL, cholangiocarcinoma; COAD, colon adenocarcinoma; ESCA, esophageal carcinoma; LIHC, liver hepatocellular carcinoma; PAAD, pancreatic adenocarcinoma; READ, rectum adenocarcinoma; STAD, stomach adenocarcinoma; BLCA, bladder urothelial Carcinoma; KIRC, kidney renal clear cell carcinoma; KIRP, kidney renal papillary cell carcinoma; TGCT, testicular germ cell tumor; LUAD, lung adenocarcinoma; LUSC, lung squamous cell carcinoma. $P < 0.05$ was considered statistically significant.

Fig. S3. Pairwise comparisons of ACE2 expression between cancers and adjacent normal tissues.

Pairwise comparisons in COAD (a), ESCA (b), LIHC (c), PAAD (d), STAD (e), BLCA (f), KIRC (g), KIRP (h), PRAD (i), LUAD (j) or LUSC (k). Wilcoxon matched-pairs signed rank tests or paired t tests were performed. TCGA, The Cancer

Genome Atlas; COAD, colon adenocarcinoma; ESCA, esophageal carcinoma; LIHC, liver hepatocellular carcinoma; PAAD, pancreatic adenocarcinoma; STAD, stomach adenocarcinoma; BLCA, bladder urothelial carcinoma; KIRC, kidney renal clear cell carcinoma; KIRP, kidney renal papillary cell carcinoma; PRAD, prostate adenocarcinoma; LUAD, lung adenocarcinoma; LUSC, lung squamous cell carcinoma. ns, not significant; *, P < 0.05; **, P < 0.01.

Fig. S4. Correlations between ACE2 expression and immune infiltration across 32 cancer types through TIMER.

(a) Heatmap showing the correlations between ACE2 expression and immune cell infiltration. (b) Heatmap showing the correlations between ACE2 expression and gene markers of immune cells, the x-axis represents different gene markers of immune cells. Partial_Cor, purity-adjusted partial Spearman's correlation; TAM, tumor associated macrophage; Th1, T-helper 1; Th2, T-helper 2; Tfh, follicular helper T; Th17, T-helper 17; Treg, regulatory T cell.

Table S1. The RNA and protein expressions of ACE2 in normal tissues and cells in the Human Protein Atlas.

Table S2. Immunohistochemistry results of ACE2 expressions in cancers from the pathology atlas.

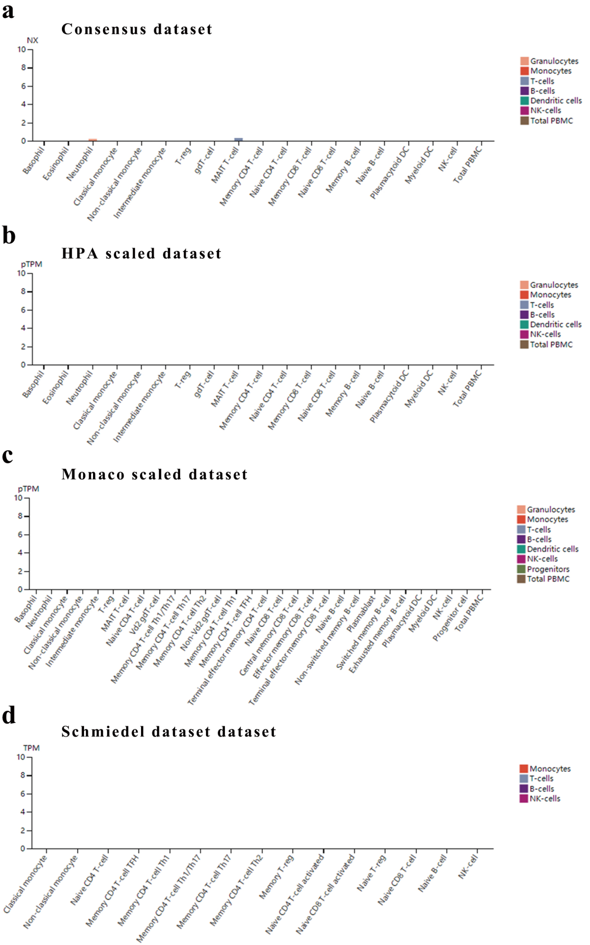
a
Consensus dataset
NX
10
8
6
4
2
0
Basophil
Eosinophil
Neutrophil
Classical monocyte
Non-classical monocyte
Intermediate monocyte
T-reg
gdT-cell
MAIT T-cell
Memory CD4 T-cell
Naive CD4 T-cell
Memory CD8 T-cell
Naive CD8 T-cell
Memory B-cell
Naive B-cell
Plasmacytoid DC
Myeloid DC
NK-cell
Total PBMC
Granulocytes
Monocytes
T-cells
B-cells
Dendritic cells
NK-cells
Total PBMC
b
HPA scaled dataset
pTPM
10
8
6
4
2
0
Basophil
Eosinophil
Neutrophil
Classical monocyte
Non-classical monocyte
Intermediate monocyte
T-reg
gdT-cell
MAIT T-cell
Memory CD4 T-cell
Naive CD4 T-cell
Memory CD8 T-cell
Naive CD8 T-cell
Memory B-cell
Naive B-cell
Plasmacytoid DC
Myeloid DC
NK-cell
Total PBMC
Granulocytes
Monocytes
T-cells
B-cells
Dendritic cells
NK-cells
Total PBMC
c
Monaco scaled dataset
pTPM
10
8
6
4
2
0
Basophil
Neutrophil
Classical monocyte
Non-classical monocyte
Intermediate monocyte
T-reg
MAIT T-cell
Naive CD4 T-cell
Vd2 gdT-cell
Memory CD4 T-cell Th1/Th17
Memory CD4 T-cell Th17
Memory CD4 T-cell Th2
Non-Vd2 gdT-cell
Memory CD4 T-cell Th1
Memory CD4 T-cell TFH
Terminal effector memory CD4 T-cell
Naive CD8 T-cell
Central memory CD8 T-cell
Effector memory CD8 T-cell
Terminal effector memory CD8 T-cell
Naive B-cell
Non-switched memory B-cell
Plasmablast
Switched memory B-cell
Exhausted memory B-cell
Plasmacytoid DC
Myeloid DC
NK-cell
Progenitor cell
Total PBMC
Granulocytes
Monocytes
T-cells
B-cells
Dendritic cells
NK-cells
Progenitors
Total PBMC
d
Schmiedel dataset dataset
TPM
10
8
6
4
2
0
Classical monocyte
Non-classical monocyte
Naive CD4 T-cell
Memory CD4 T-cell TFH
Memory CD4 T-cell Th1
Memory CD4 T-cell Th1/Th17
Memory CD4 T-cell Th17
Memory CD4 T-cell Th2
Memory T-reg
Naive CD4 T-cell activated
Naive CD8 T-cell activated
Naive T-reg
Naive CD8 T-cell
Naive B-cell
NK-cell
Monocytes
T-cells
B-cells
NK-cells

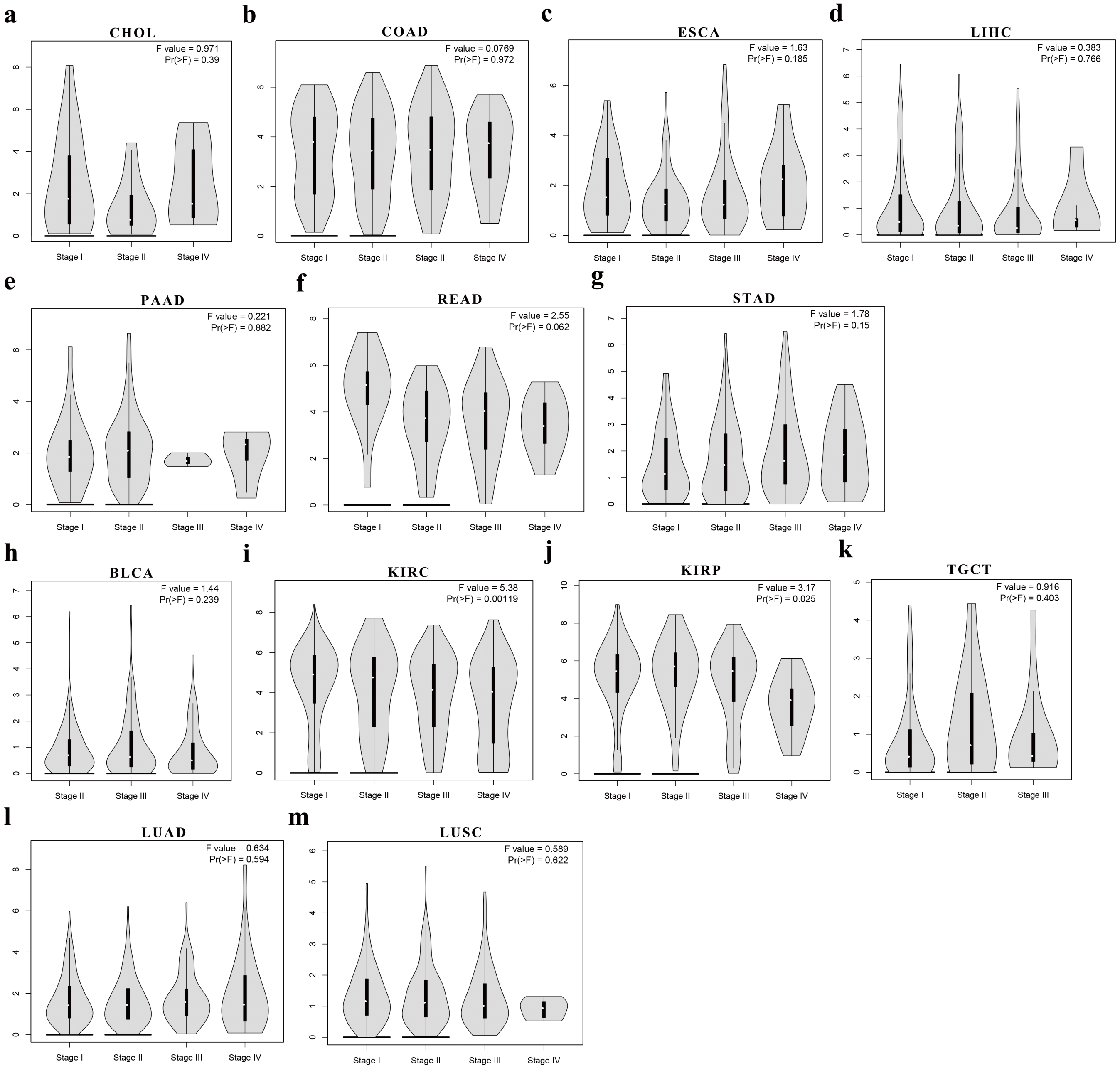

a
CHOL
F value = 0.971
Pr(>F) = 0.39
Stage I
Stage II
Stage IV
b
COAD
F value = 0.0769
Pr(>F) = 0.972
c
ESCA
F value = 1.63
Pr(>F) = 0.185
d
LIHC
F value = 0.383
Pr(>F) = 0.766
e
PAAD
F value = 0.221
Pr(>F) = 0.882
f
READ
F value = 2.55
Pr(>F) = 0.062
g
STAD
F value = 1.78
Pr(>F) = 0.15
h
BLCA
F value = 1.44
Pr(>F) = 0.239
i
KIRC
F value = 5.38
Pr(>F) = 0.00119
j
KIRP
F value = 3.17
Pr(>F) = 0.025
k
TGCT
F value = 0.916
Pr(>F) = 0.403
l
LUAD
F value = 0.634
Pr(>F) = 0.594
m
LUSC
F value = 0.589
Pr(>F) = 0.622
Stage I
Stage II
Stage III
Stage IV

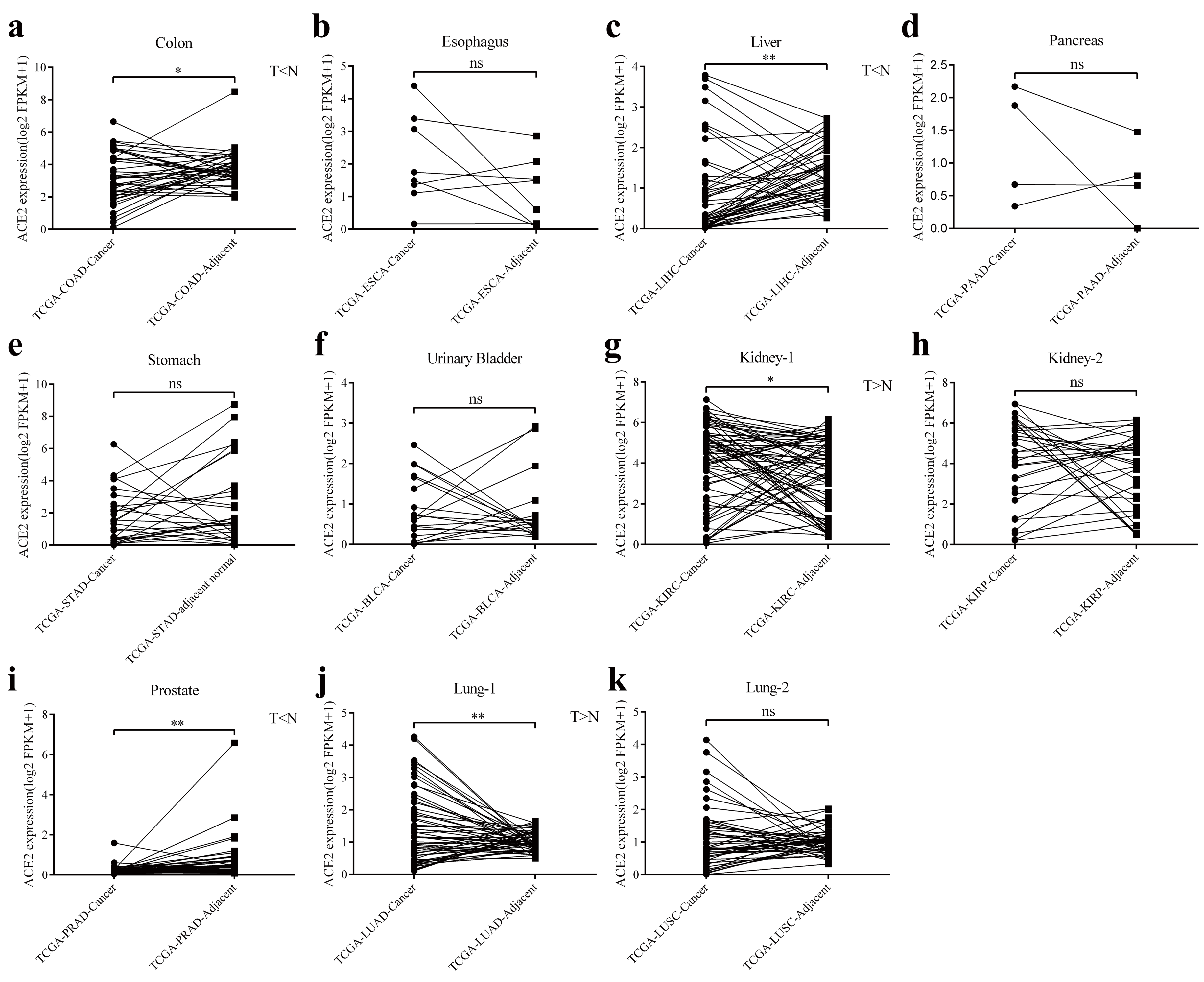
a
Colon
T<N
b
Esophagus
c
Liver
T<N
d
Pancreas
e
Stomach
f
Urinary Bladder
g
Kidney-1
T>N
h
Kidney-2
i
Prostate
T<N
j
Lung-1
T>N
k
Lung-2
ACE2 expression(log2 FPKM+1)
TCGA-COAD-Cancer
TCGA-COAD-Adjacent
TCGA-ESCA-Cancer
TCGA-ESCA-Adjacent
TCGA-LIHC-Cancer
TCGA-LIHC-Adjacent
TCGA-PAAD-Cancer
TCGA-PAAD-Adjacent
TCGA-STAD-Cancer
TCGA-STAD-adjacent normal
TCGA-BLCA-Cancer
TCGA-BLCA-Adjacent
TCGA-KIRC-Cancer
TCGA-KIRC-Adjacent
TCGA-KIRP-Cancer
TCGA-KIRP-Adjacent
TCGA-PRAD-Cancer
TCGA-PRAD-Adjacent
TCGA-LUAD-Cancer
TCGA-LUAD-Adjacent
TCGA-LUSC-Cancer
TCGA-LUSC-Adjacent

Table S1. The RNA and protein expression of ACE2 in normal tissues and cells in the Human Protein Atlas

Tissues	Cells	Tissue RNA	Cell Protein
adipose tissue		4.5	
	adipocytes		nd
adrenal gland		0.4	
	glandular cells		low
amygdala		0.2	
appendix		0.8	
	glandular cells		nd
	lymphoid tissue		nd
B-cells		0	
basal ganglia		0.2	
bone marrow		0	
	hematopoietic cells		nd
breast		2.3	
	adipocytes		nd
	glandular cells		nd
	myoepithelial cells		nd
Brochus			
	respiratory epithelial cells		nd
caudate			
	glial cells		nd
	neuronal cells		nd
cerebellum		0.2	
	cells in granular layer		nd
	cells in molecular layer		nd
	purkinje cells		nd
	endothelial cells		nd
cerebral cortex		0.2	
	endothelial cells		nd
	glial cells		nd
	neuronal cells		nd
	neuropil		nd
cervix, uterine		0.4	
	glandular cells		nd
	squamous epithelial cells		nd
colon		49.1	
	endothelial cells		nd
	glandular cells		low
	peripheral nerve/ganglion		nd
corpus callosum		0.2	
dendritic cells		0	
ductus deferens		2.3	

duodenum		46	
	glandular cells		high
endometrium		0.4	
	cells in endometrial stroma		nd
	glandular cells		nd
epididymis		2.7	
	glandular cells		nd
esophagus		1.2	
	squamous epithelial cells		nd
fallopian tube		0.6	
	glandular cells		nd
gallbladder		16.4	
	glandular cells		high
granulocytes		0.2	
heart muscle		10.5	
	myocytes		nd
hippocampal formation		0.2	
	glial cells		nd
	neuronal cells		nd
hypothalamus		0.1	
kidney		23.2	
	cells in glomeruli		nd
	cells in tubules		high
liver		1.2	
	bile duct cells		nd
	hepatocytes		nd
lung		0.8	
	macrophages		nd
	pneumocytes		nd
lymph node		0.6	
	germinal center cells		nd
	non-germinal center cells		nd
midbrain		0.2	
monocytes		0	
nasopharynx			
	respiratory epithelial cells		nd
natural killer cells		0	
olfactory region		0.2	
oral mucosa			
	squamous epithelial cells		nd
ovary		1.3	
	follicle cells		nd
	ovarian stroma cells		nd

pancreas		1.6	
	exocrine glandular cells		nd
	islets of Langerhans		nd
parathyroid gland		0	
	glandular cells		nd
pituitary gland		0.2	
placenta		1	
	decidual cells		nd
	trophoblastic cells		nd
pons and medulla		0.2	
prostate		0.5	
	glandular cells		nd
rectum		1.3	
	glandular cells		low
retina		0.2	
salivary gland		1.1	
	glandular cells		nd
seminal vesicle		1.2	
	glandular cells		low
skeletal muscle		0.7	
	myocytes		nd
skin		0.2	
	fibroblasts		nd
	keratinocytes		nd
	Langerhans		nd
	melanocytes		nd
	epidermal cells		nd
small intestine		122	
	glandular cells		high
smooth muscle		0.3	
	mooth muscle cells		nd
soft tissue			
	chondrocytes		nd
	fibroblasts		nd
	peripheral nerve		nd
spinal cord		0.2	
spleen		0.2	
	cells in red pulp		nd
	cells in white pulp		nd
stomach		0.5	
	glandular cells		nd
substantia nigra		0.2	
T cells		0.3	
testis		17.9	

	cells in seminiferous ducts		high
	leydig cells		high
thalamus		0.2	
thymus		0.2	
thyroid gland		4.5	
	glandular cells		nd
tongue		0.5	
tonsil		0.2	
	germinal center cells		nd
	non-germinal center cells		nd
	squamous epithelial cells		nd
total PBMC		0	
urinary bladder		0.4	
	urothelial cells		nd
vagina		0.9	
	squamous epithelial cells		nd

nd, not detected; PBMC, peripheral blood mononuclear cells.

Table S2. Immunohistochemistry results of ACE2 expression in cancers from the pathology atlas

Cancer type	Antibody	High	Medium	Low	nd
Breast cancer	HPA000288	0	0	1	8
	CAB026174	0	0	0	9
Carcinoid	HPA000288	0	0	0	4
	CAB026174	0	0	0	4
Cervical cancer	HPA000288	0	0	0	11
	CAB026174	0	0	1	11
Colorectal cancer	HPA000288	4	7	0	1
	CAB026174	1	1	2	7
Endometrial cancer	HPA000288	0	0	0	9
	CAB026174	0	0	0	12
Glioma	HPA000288	0	0	0	12
	CAB026174	0	0	0	11
Head and neck cancer	HPA000288	0	0	1	3
	CAB026174	0	0	0	4
Liver cancer	HPA000288	0	2	0	5
	CAB026174	1	1	2	8
Lung cancer	HPA000288	0	0	1	11
	CAB026174	0	0	0	9
Lymphoma	HPA000288	0	0	0	12
	CAB026174	0	0	0	12
Melanoma	HPA000288	0	0	0	11
	CAB026174	0	0	0	12
Ovarian cancer	HPA000288	0	2	0	10
	CAB026174	0	0	0	10
Pancreatic cancer	HPA000288	0	4	1	7
	CAB026174	0	0	3	8
Prostate cancer	HPA000288	0	0	0	11
	CAB026174	0	0	0	12
Renal cancer	HPA000288	5	3	2	1
	CAB026174	6	5	1	0
Skin cancer	HPA000288	0	0	0	10
	CAB026174	0	0	0	12
Stomach cancer	HPA000288	0	4	3	5
	CAB026174	0	1	1	7
Testis cancer	HPA000288	0	2	0	10
	CAB026174	1	0	0	11
Thyroid cancer	HPA000288	0	0	1	3
	CAB026174	0	0	0	4
Urothelial cancer	HPA000288	0	0	2	10
	CAB026174	0	1	0	10

nd, not detected.
